# Misdiagnosis of pulmonary tuberculosis and associated factors in peripheral laboratories: a retrospective study, Addis Ababa, Ethiopia

**DOI:** 10.1186/s13104-018-3414-6

**Published:** 2018-05-11

**Authors:** Daniel Melese Desalegn, Kumera Terfa Kitila, Hanna Mekonnen Balcha, Chalachew Sisay Gebeyehu, Yohannes W. Kidan, Kassayenew Amare, Daniel Dejene, Merone Seifu, Addis Zewdie, Abiyot Tenna, Tinsae Kidanemariam Hailu, Boja Dufera Taddese, Abrham Tesfaye Bika

**Affiliations:** 1 Addis Ababa Public Health Research and Emergency Management Core Process, Addis Ababa City Administration Health Bureau, Addis Ababa, Ethiopia; 2 Ethiopian Public Health Institute (EPHI), Addis Ababa, Ethiopia

**Keywords:** External quality assessment, ZN sputum smear microscopy, Addis Ababa

## Abstract

**Objective:**

Sputum smear microscopy reading errors are likely to result in failure to detect persons with infectious TB. This study was intended to review misdiagnosis of pulmonary TB and associated factors in peripheral laboratories.

**Results:**

During the study period 1033 (10.5%) sputum smear positive and 8783 (89.5%) smear negative slides were reported by peripheral laboratories. The slides were re-read by the central referral laboratories (CRLs) as the reference standard reading. Of 1033 positive slides reported by peripheral laboratories, 25 (2.4%) were false positive. Out of 8783 smear negative slides reported by peripheral laboratories, 35 (0.4%) were false negative. The sensitivity, specificity, positive predictive value and negative predictive value of peripheral laboratories were 96.64, 99.72, 97.58, and 99.61% respectively. The peripheral laboratories and CRLs have an observed agreement (Po) of 0.9939. Of 135 peripheral laboratories, 93 (68.9%) read negative and positive slides correctly, 49 (36.3%) did not have lens cleaning tissue papers, 11 (8.1%) lacked frosted slides, and 14 (10.4%) had shortage of reagents. As conclusions, the peripheral laboratories and CRLs had high agreement for sputum smear microscopy reading. However, a few TB cases were misdiagnosed despite having the disease; these individuals might continue to spread the infection in the community.

## Introduction

Laboratory services continue playing critical role in diagnosing TB and treatment monitoring [1, 2]. However, it requires strong quality assurance (QA) systems be in place [3–6]. In contrary, reading errors in ZN-stained sputum smear microscopy are likely to result in failure to detect persons with infectious TB, consequently continues to spread infection in the community, or may unnecessary treated for non TB cases [5–7]. Moreover, in adequately training, erratic reagent, supplies, and poor equipment maintenance inflate the problems.

Accurate and reliable TB microscopy result relies on external quality assessment (EQA) programs that support, train, and monitor testing performance of individual laboratories [3, 8]. External quality assessment comprises proficiency testing, blind-rechecking of samples and on-site evaluation [7–9]. In study setting, blind-rechecking programs is crucial EQA method in assessing sputum smear microscopy reading performance of the laboratory [7, 8]. It provides an opportunity to assess quality performance elements including specimen quality, smear size and thickness, and quality of staining. All these information may be very useful to assessing possible reasons for false positive or false negative results, and implementing plans for retraining and corrective action [3, 9, 10].

Information concerning the performance of EQA in private and public laboratories in Addis Ababa is very limited; hence we reviewed 135 public and private health facilities’ ZN sputum smear microscopy EQA performance, to determine misdiagnosis of pulmonary TB cases and associated factors in private and public health facilities in Addis Ababa, Ethiopia.

## Main text

### Methods

Retrospective review of EQA records in 135 public and private laboratories in Addis Ababa Ethiopia was conducted, from October, 2014 to March, 2016. These peripheral laboratories provide TB diagnostic services and they participate in ZN smear microscopy Regional External Quality Assurance Scheme (REQAS). This study compared the results of peripheral laboratories’ ZN-stained sputum smear microscopy reading performances with CRLs as a gold standard.

#### Study subjects

One hundred thirty five peripheral laboratories (45 privates and 90 public) providing TB diagnostic service to patients in Addis Ababa were considered for the current study.

#### Data sources and sampling

Data were collected from 135 health facilities in Addis Ababa. The health facilities’ ZN-stained sputum smear microscopy EQA performances and onsite EQA records were used as data source.

#### Inclusion and exclusion criteria

All peripheral laboratories having complete EQA performance records in the CRLs data storage, from the years 2014–2016 were included. However, TB laboratory results performed by other than ZN technique were excluded.

#### Data management and statistical analysis

The collected data were checked for completeness and consistency and entered into using Epi-info software. The data were coded and analysed using SPSS version 20.0 (SPSS Inc. Chicago, USA) software. ZN-stained sputum smear microscopy reading agreement between the peripheral microscopy centers and the CRLs were measured by using Kappa (K). Inter-observer variability was assessed on the basis of κ-values, with kappa coefficient: 0.81–1 = almost perfect, 0.61–0.8 = substantial, 0.41–0.60 = moderate, 0.21–0.40 = fair, 0.01–0.20 = slight and ≤ 0 = poor agreement between slide readers [11]. Sensitivity, specificity, PPV and NPV of the peripheral laboratories were calculated against the final readings of the CRLs as a gold standard. Bivariate and Multivariate logistic regression model using odds ratio (OR) at 95% confidence interval (CI) were calculated. P value less than 0.05 were taken as statistically significant.

#### Data quality assurances

Before extracting data from records, data collectors were adequately trained and the collected data were checked for completeness by data collectors and authors. Data collection process was supervised and monitored by the principal investigator.

#### Operational definition

- Central referral laboratories (CRLs): Regional and intermediate level (Hospital) laboratories serving as ZN smear microscopy a quality assurance (REQAS) centers for peripheral laboratories.
- Peripheral laboratories: Laboratories located at a Health Center or private Hospital and different level of Clinics, providing ZN smear microscopy diagnostic services.
- Discordant slides: Positive slides read as negative or vice versa.

### Result

#### ZN-stained sputum smears microscopy reading agreement

During the study period 135 peripheral laboratories were involved in REQAS. Among these, 45 (33.3%) were private and the remaining 90 (66.7%) public health facilities. Of 135 peripheral laboratories, 93 (68.9%) correctly read negative and positive sputum smear slides. The remaining 42 (31.1%) laboratories misread at least one positive sputum smear slides as negative or vice versa. Out of 42 health peripheral laboratories reported false reading results, 23 (54.8%) were public and the remaining 19 (45.2%) were private’ laboratories. However, there was no statistically significant difference in sputum smear microscopy false reading results between public and private laboratories (P value > 0.05).

Of 9816 ZN-stained sputum smear slides were collected from the peripheral laboratories for re-checking, 1033 (10.5%) were reported as positive, and the reaming 8783 (89.5%) were reported as negative by peripheral laboratories. The slides were re-read by the CRLs, which were considered as the reference standard reading. Of 1033 sputum smear slides reported as positive by peripheral laboratories, 1008 (97.6%) were true positive, the remaining 25 (2.4%) were false positive. Out of 8783 sputum smear slides reported as negative by peripheral laboratories, 8748 (99.6%) were true negative, the remaining 35 (0.4%) were false negative. The overall discordant rate between peripheral laboratories and CRLs was 60 (0.61%) (Table 1).

**Table 1:** Sputum smears microscopy reading agreement between peripheral laboratories and central referral laboratories in Addis Ababa, Ethiopia

Peripheral laboratories smear results (test value)	Central referral laboratories results (as true value)		Total
	Positive	Negative	
Positive	1008	25	1033
Negative	35	8748	8783
Total	1043	8773	9816

The study has shown an observed agreement (Po) of 0.9939, expected agreement (Pe) of 0.8109 and calculated kappa value was 0.97, which indicated almost perfect agreement. The sensitivity, specificity, PPV and NPV of peripheral laboratories in ZN-stained sputum reading was 96.64, 99.72, 97.58 and 99.6% respectively.

#### Smear quality

None of the peripheral laboratories assessed met sputum smear quality standards. Of 9816 collected sputum smear slides from the peripheral laboratories, 3475 (35.4%) specimen quality, 3316 (33.8%) evenness, 3004 (30.6%) smear size and 2817 (28.7%) labeling were found as poor. Moreover, 3846 (39.2%) smears were prepared with inappropriate thickness, and 3029 (30.9%) smears were unacceptable staining background cleanness. Compared to the performance of public and private laboratories for each of the quality indices, of 3168 sputum smear slides collected from the private laboratories, 1163 (36.7%) specimen quality, 1042 (32.9%) evenness, 991 (31.3%) smear size, 916 (28.9%) labeling, 1291 (40.8%) thickness, and 1039 (32.8%) staining background were unacceptable. Of 6648 slides collected from the public laboratories, 2312 (34.8%) specimen quality, 2274 (34.2%) evenness, 2013 (30.3%) smear size and 1901 (28.6%) labeling, 2552 (38.4%) thickness, and 2053 (30.9%) staining background were found as poor.

#### Tuberculosis laboratory facilities and infrastructures

On-site evaluation of EQA was conducted in 135 peripheral laboratories to assess the status of TB laboratory infrastructures and factors affecting the quality of ZN-stained sputum microscopy diagnosis. The entire assessed peripheral laboratories had functional light microscope, 104 (77.0%) had separate work station for smearing, staining, microscopy examination and recording. Of the total TB laboratories assessed, 79 (58.5%) had clean, separate and ventilated TB laboratory room, 127 (94.1%) had regular water supply and 131 (97.0%) had back up electric power supply (Table 2).

**Table 2:** TB laboratory diagnostic facilities and infrastructure assessment, in Addis Ababa, Ethiopia

Variables	Number	Percent
Separate TB laboratory room		
Yes	79	58.5
No	56	41.5
Separate area for TB laboratory works		
Yes	104	77.0
No	31	23.0
Regular electric power supply		
Yes	131	97
No	19	14.1
Regular water supply		
Yes	127	94.1
No	8	5.9
Runn IQC slides parallel with routine tasks		
Yes	38	28.1
No	97	71.9
SOPs, guideline and manuals		
Yes	107	79.3
No	28	20.7
Functional light microscopes		
Yes	135	100
No	0	0.0
ZN-staining reagents		
Yes	121	89.6
No	14	10.4
Lens cleaning tissue paper		
Yes	49	36.3
No	86	63.7
IQC reagents		
Yes	26	19.3
No	109	80.7
Good storage condition for reagents		
Yes	82	60.7
No	53	39.3
Checking quality of sputum		
Yes	76	56.3
No	59	43.7
Always carbol-fuchsin filtered before use		
Yes	78	57.8
No	57	42.2
Having news slides (frosted slide)		
Yes	124	91.9
No	11	8.1
Re-used sputum container		
Yes	0	0.0
No	135	100
Having personal protective equipment		
Yes	135	100
No	0	0.0
Having functioning incinerator		
Yes	135	100
No	0	0.0
Staff received training on ZN sputum smears microscopy		
Yes	103	76.3
No	32	23.7
Having standard TB result registration log-book		
Yes	109	80.7
No	26	19.3
Received EQA feedback		
Yes	124	91.9
No	11	8.1

Shortage of supplies including all type of reagents (Carbol-fuchsin, methylene blue and Acid alcohol), lens tissue; frosted slides, internal quality control (IQC) reagents were identified in most peripheral laboratories. Of 135 peripheral laboratories, 49 (36.3%) did not have lens cleaning tissue papers, 11 (8.1%) lacked frosted slides, 14 (10.4%) had shortage of ZN-staining reagents, and nearer to half (42.2%) did not filter 1% carbol-fuchsin during staining of sputum smears. However, all peripheral laboratories used the appropriate personal protective equipment (PPE) when performing TB laboratory procedures, and they used incinerator to dispose sputum, and other sputum contaminated solid wastes (Table 2).

#### Quality assurance

There were gaps in having valid internal quality control materials in all assessed laboratories. Among these, 38 (28.1%) laboratories prepare smears from known positive and negative sputum as internal quality control to check reagents quality before staining patient sample. About 59 (43.7%) did not check sputum quality before processing. Among 135 visited laboratories, 109 (80.7%) had standard TB laboratory request form and result log book, 107 (79.3%) had TB national guideline; manual, SOPs and other reference materials for smear microscopy technique. More than half (76.3%) of the peripheral laboratories’ staffs members were trained on sputum smear microscopy (Table 2). From those who took training, 73 (70.9%) were from public health facilities.

#### Factors affecting sputum smear microscopic reading

In binary logistic regression, false reading of sputum smear microscopy had significant association with checking the quality of sputum before smear preparation, having separate TB laboratory room and filtering carbol-fuchsin before staining sputum smear (P value < 0.05). However, in multivariate logistic regression analysis, false reading had no significant association with these factors (P value > 0.05) (Table 3).

**Table 3:** Factors affecting for ZN sputum smears microscopy reading in peripheral diagnostic facilities, Addis Ababa, Ethiopia

Variables	False reading		COR (95% CI)	P value	AOR (95% CI)	P value
	No	Yes				
Separate TB laboratory room						
Yes	31	29	1			
No	25	50	2.14 (1.06, 4.30)	0.033*	0.46 (0.74, 2.89)	0.41
Training on ZN microscopy						
Yes	14	46	1			
No	18	57	0.96 (0.43, 2.14)	0.93		
Cheek quality of sputum						
Yes	32	28	1			
No	27	48	2.03 (1.02, 4.06)	0.04*	0.63 (0.12, 3.28)	0.59
Use new slides						
Yes	6	54	1			
No	5	70	1.56 (0.45, 5.37)	0.49		
Filter reagent before use						
Yes	31	29	1			
No	26	49	2.02 (1.02, 4.04)	0.048*	1.54 (0.14, 4.17)	0.73
Prepared and use IQC						
Yes	25	10	1			
No	84	16	0.47 (0.32, 1.79)	0.53		
Having lenses tissue						
Yes	20	15	1			
No	66	34	0.68 (0.29, 1.17)	0.13		
Good reagent storage condition						
Yes	34	66	1			
No	19	16	0.43 (0.28, 1.15)	0.12		
Received feed back						
Yes	6	69	1			
No	5	55	0.96 (0.28, 3.30)	0.94		
Owner ship						
Private	26	19	1			
Government	67	23	0.87 (0.42, 1.80)	0.47		
Having ZN reagents						
Yes	57	4	1			
No	63	11	2.49 (0.87, 12.29)	0.08		

* Statistically significant P < 0.05

### Discussion

In the current study, sputum smear microscopy reading agreements between the peripheral laboratories with the CRLs were evaluated, using CRLs as reference standard. The sensitivity, specificity, PPV and NPV of the peripheral laboratories were 96.64, 99.72, 97.58 and 99.61%, respectively, which is comparable to 95, 99.7, 93.3 and 99.7% respectively reported in Ethiopia and 91.3, 98.9, 92.2 and 98.8% respectively reported in Argentine [12, 13]. In contrast, it is higher than others studies [14–16]. In present study overall sputum smear reading agreement between the peripheral diagnostic laboratories and CRLs was 99.4%, which is comparable to 99.5% reported in west Amhara Region [17] and 99.7%, reported Lima, Peru [18]. However, it is higher compared to 96.8% reported in south Ethiopia [19] and 87% reported in Addis Ababa [16] and 89.2% report in Tanzania [20]. Although, the proportion of sputum smear microscopy reading agreement of 99.4% achieved is higher than acceptable performance of > 80% [7, 10, 21], still disagreement of 0.6% may indicate a need to improve the quality of TB microscopic reading.

In this study overall discordant (false reading) results of sputum microscopy reported by the peripheral laboratories was 60 (0.6%), which is lower than the previous reports of 7.8% in Argentine, 3.5% in west Amhara Region, 3.2% in southern Ethiopia, 3.3% in India, and 5.5% in eastern part of Ethiopia [13, 17, 19, 22, 23]. It is higher than study reported 0.2% in Ethiopia and 0.3% in Taiwan [12, 24]. These variations might be due to sputum smear microscopy reading is highly dependent on the training, diligence of microscopist and laboratory supplies. The other possible reason, unfiltered fuchsin crystals reagents, weak decoloration of AFB might affect the quality of ZN smear microscopy reading and lead to false reading results [16, 20, 26].

In the present study specimen quality, evenness, smears size, inappropriate labeling, thickness, and smears with unacceptable staining background cleanness were 35.4, 33.8, 30.6, 28.7, 39.2 and 31.5% respectively, which is relatively lower than other studies [24–26]. Specimen container, sample transport condition, specimen quality, labeling, smearing, and staining technique including failure to filter carbol fuchsin are some of the factors that compromise ZN sputum smears quality performance indices [20, 26].

In the current study 79 (58.5%) of the peripheral laboratories having clean, separate and ventilated TB laboratory room, which is higher than 18.2–42% previously reported in other studies [15, 16, 27, 28]. Unventilated TB laboratory room can create unfavorable working environment, which might affect the overall quality of sputum smear microscopy services from pre-analytical to post analytical phases. In addition, this may create a chance of cross contamination [2, 29].

Regarding TB laboratory safety issue, all the peripheral laboratories used the appropriate PPE and disposed sputum and other sputum contaminated solid wastes appropriately. This finding was higher when compared with study conducted in Malawi, which reported most hospitals’ laboratories didn’t wear white coat, face mark, protective apron and soap for washing hand [27]. The difference might be due to availability of PPE, professionals’ attitude towards PPE. A poor laboratory safety practice does not only put the laboratory workers at risk of infection, but also the patients and any other person accessing the laboratory services.

### Conclusion

In the present study peripheral laboratories and CRLs had high agreement for ZN sputum smear microscopy reading. However, a few TB cases were misdiagnosed despite having the disease; these individuals might continue to spread the infection in the community. This indicates the needs to improve ZN-stain sputum smear microscopy reading to attain the end TB strategy.

## Limitations of the study

- Results of this study depend only on the TB-EQA record review. Therefore, not illustrate providers view.

## Abbreviations

AFB: acid fast bacillus (or bacilli)
AOR: adjusted odds ratio
CI: confidence interval
CRLs: central referral laboratories
EQA: external quality assessment
NPV: negative predictive value
COR: crude odds ratio
SPSS: Statistical Package for Social Sciences
PPV: positive predictive value
TB: tuberculosis
WHO: World Health Organization
ZN: Ziehl–Neelsen stain
REQAS: Regional External Quality Assurance Scheme
